# Impact of the time interval between end of induction and autologous hematopoietic transplantation in newly diagnosed patients with multiple myeloma

**DOI:** 10.1038/s41409-022-01835-y

**Published:** 2022-10-06

**Authors:** Charalampos Charalampous, Utkarsh Goel, Morie Gertz, Martha Lacy, Angela Dispenzieri, Suzanne Hayman, David Dingli, Francis Buadi, Prashant Kapoor, Taxiarchis Kourelis, Rahma Warsame, William J. Hogan, Shaji Kumar

**Affiliations:** Division of Hematology, Department of Internal Medicine, Mayo Clinic, Rochester, MN USA

**Keywords:** Risk factors, Myeloma

## Abstract

Multiple Myeloma patients eligible for autologous hematopoietic transplantation (AHT) typically receive 3–6 cycles of induction therapy before transplant. The last induction cycle is completed 2–4 weeks prior to mobilization. We evaluated the impact of the time interval between end of induction and AHT on progression-free survival (PFS) and overall survival (OS). A total of 1055 patients who underwent AHT were identified. The median time to transplant (TTT) was 33 days (27–42 quartile range). Patients with less than 33 days of TTT had significantly prolonged PFS (35.6 vs. 32.1 months, *p* < 0.03) but non-significant OS differences compared to those with more than 33 days. Quartile comparisons showed that patients in the 1st quartile (less than 27 days) had significantly prolonged PFS (36.7 vs. 30.9 months, *p* < 0.01) compared to the 4th quartile group (more than 42 days). In a subgroup analysis of patients with partial or worse biochemical response prior to transplant, patients in the 1st quartile had significantly prolonged PFS (37.7 vs. 28.7 months, *p* < 0.04) compared to the 4th quartile group. In conclusion, we showed that a prolonged TTT is associated with inferior outcomes compared to tighter chemotherapy schedules. This finding was especially prevalent in patients with partial response at induction.

## Introduction

Multiple myeloma (MM) is a monoclonal malignancy of terminally differentiated plasma cells and is the second most common hematologic malignancy in the United States [1, 2]. Recent advances in the therapeutic regimens and the consistent use of autologous stem cell transplant (ASCT) for eligible patients have significantly improved survival outcomes over the last 2 decades [3, 4]. The role of transplant has been recently challenged by the advent of newer drugs and combinations capable of effectively controlling the disease and achieving comparable outcomes without consolidative transplantation [5]. This effort has been mainly driven by the toxicity of transplant-related procedures (e.g., infection), albeit significantly minimized in duration and typically reversible [6]. Still, many clinical trials have shown a significant survival benefit for ASCT vs. solely drug management; nearly one-third of patients achieve a complete response (CR) with an estimated median PFS from diagnosis of 18 months to 2 years without any additional treatment [7, 8]. After transplant, most patients receive maintenance therapy with either lenalidomide or a bortezomib-based regimen [9].

Patients eligible for upfront transplant typically receive an induction regimen for 3–6 cycles before proceeding to a high-dose melphalan course and autologous stem cell infusion [10]. Typically, the last induction cycle is completed 3–4 weeks before stem cell mobilization and apheresis [11]. While using CXCR antagonists like plerixafor reduces delays with apheresis, other logistical issues can increase the gap between the end of induction and the start of ASCT [12]. Subsequently, patients receive high-dose ablative chemotherapy, and the stem cells are re-infused [13]. Although many risk factors (e.g., age, obesity, prior radiation exposure) have been identified to contribute to transplantation outcomes [14–16], it is unclear if worsening of disease during the drug-free period between the last chemotherapy date and the date of stem cell infusion predicts for high-risk disease and poorer clinically relevant outcomes after transplant. In this study, we wanted to evaluate the impact of this chemotherapy-free period on progression-free survival (PFS) and overall survival (OS) post ASCT.

## Patients and methods

### Patients

This retrospective cohort study included all newly diagnosed MM (NDMM) patients diagnosed from 2004 to 2018 who were seen in Mayo Clinic and underwent upfront ASCT within a year from diagnosis. Patients that progressed during induction therapy were excluded from the study. In addition, we excluded patients who received cytoxan pulsing prior to mobilization because that would introduce an artificially prolonged time to transplant compared to standard protocol. To ensure homogeneity in our cohort, we excluded all patients that received maintenance therapy or other chemotherapy regimens after their stem cell collection.

First, we assessed the duration of each patient’s induction regimen. The date of last chemotherapy was noted for the patients with an exact date available. We also collected the dosing of the conditioning regimen. We then determined the biochemical response achieved in the pre-transplant evaluation, per the Internation Myeloma Working Group response criteria (IMWG). Approval for this study was obtained from the Mayo Clinic Institutional Review Board, and informed consent was obtained from all patients for review of their medical records.

### Statistical analysis

Baseline clinical characteristics were collected for the entire cohort, and comparisons were made using the Chi-squared test for categorical values. FISH stratification was based on the mSMART model and del17p, t(4;14), t(14;16), t(14;20), gain1q abnormalities were considered as high-risk [17]. We subgrouped patients into 2 groups based on the median time to transplant (TTT), calculated from the last chemotherapy date to the date of stem cell infusion. The end-points of the study were PFS, measured from the date of stem cell infusion to biochemical progression or intensification of treatment, and OS, measured from the date of stem cell infusion to death from any cause with censoring performed at the time of the last contact. A Kaplan-Meier model was used to estimate median PFS and OS and for image production. Statistical significance in PFS and OS for all tests performed was determined using the 2-sided log-rank test. The Cox proportional hazards model was utilized for multivariable analyses, which included known risk factors such as high-risk FISH, age at diagnosis, and response at induction to test for retained prognostic significance. All statistical analyses were performed using R version 4.1.1 (R Foundation for Statistical Computing, https://www.R-project.org/). A *P*-value < 0.05 was used to determine statistical significance.

## Results

A total of 1055 patients who had an autologous stem-cell transplant within a year of MM diagnosis were identified. The median age at the time of diagnosis was 61.4 years, and 332 (35.7%) patients had a high-risk FISH abnormality. Among the entire cohort, 461 (43.8%) patients were mobilized with G-CSF alone, and 588 (55.9%) had plerixafor added. In terms of conditioning, 893 (84.6%) of patients received melphalan 200 mg/m^2^, with the remaining patients [112 (10.6%)] receiving either reduced dose or other melphalan regimens, [50 (4.7%)]. The median progression-free survival (PFS) and overall survival (OS) following the date of stem cell infusion were 33.4 (95% CI = 30.8–36.4) and 122.3 (95% CI = 112.6–135.7) months, respectively. Table 1 summarizes the baseline clinical characteristics of the cohort.

**Table 1:** Baseline characteristics of the entire cohort.

	Less or equal to 33 days (*N* = 534)	More than 33 days (*N* = 521)	Total (*N* = 1055)	*p*-value
**Age**				0.029
Mean	61.1	60	60.6	
Median	62.2	60.9	61.4	
Range	23.9–75.8	28.7–76.9	23.9–76.9	
**Race**				0.737
Black	8 (1.5%)	12 (2.3%)	20 (1.9%)	
Other	19 (3.6%)	22 (4.2%)	41 (3.9%)	
Unknown	8 (1.5%)	8 (1.5%)	16 (1.5%)	
White	499 (93.4%)	479 (91.9%)	978 (92.7%)	
**High-risk FISH**				0.029
No	289 (61.0%)	310 (67.8%)	599 (64.3%)	
Yes	185 (39.0%)	147 (32.2%)	332 (35.7%)	
**Mobilization method**				0.205
G-CSF	223 (41.8%)	238 (45.9%)	461 (43.8%)	
Plerixafor added	308 (57.7%)	280 (54.1%)	588 (55.9%)	
**Conditioning regimen**				0.002
Full (Mel 200 mg/m^2^)	470 (88.0%)	423 (81.2%)	893 (84.6%)	
Reduced (Mel 140 mg/m^2^)	49 (9.2%)	63 (12.1%)	112 (10.6%)	
Other	15 (2.8%)	35 (6.7%)	50 (4.7%)	
**Biochemical response at induction**				0.043
CR	96 (18.0%)	91 (17.5%)	187 (17.7%)	
VGPR	215 (40.3%)	180 (34.5%)	395 (37.5%)	
PR	200 (37.5%)	211 (40.5%)	411 (39.0%)	
Stable disease	22 (4.1%)	39 (7.5%)	61 (5.8%)	

*FISH* Fluorescence in situ hybridization, *G-CSF* Granulocyte colony-stimulating factor, *Mel* Melphalan.

**Fig. 1:**
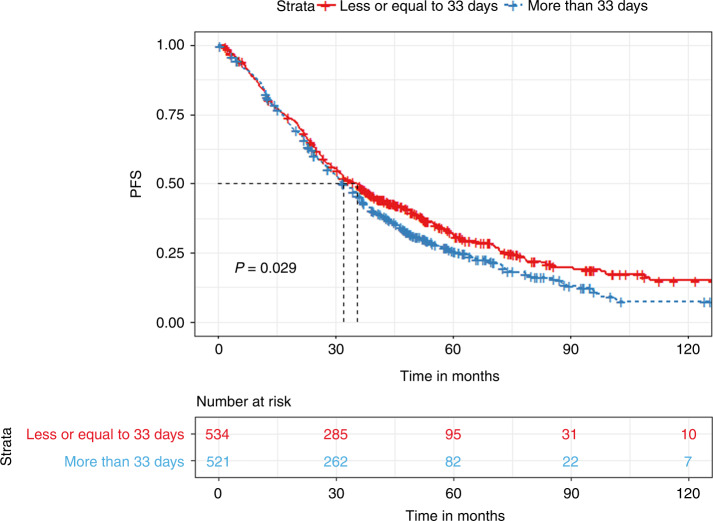
Kaplan Meier plot comparing PFS based on median TTT. Median PFS, 35.6 vs. 32.1 months, *p* < 0.03.

**Fig. 2:**
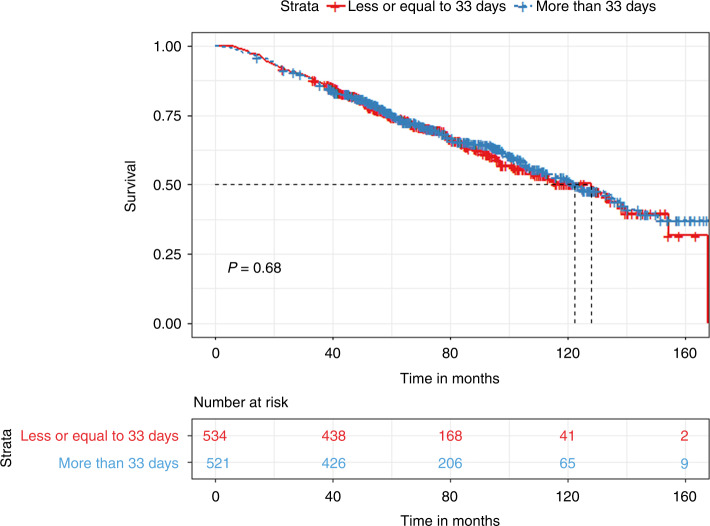
Kaplan Meier plot comparing OS based on median TTT. Median OS, 128 vs. 122.2 months, *p* = 0.68.

**Fig. 3:**
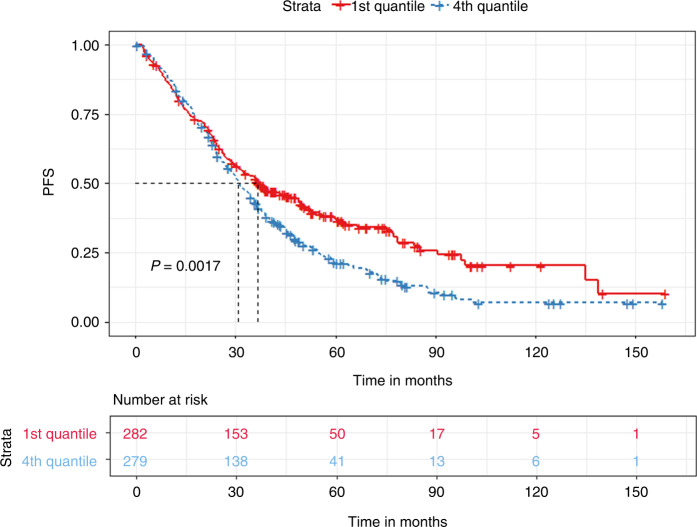
Kaplan Meier plot comparing PFS between the 1st and the 4th quartile for TTT. Median PFS, 36.7 vs. 30.9 months, *p* < 0.01.

**Fig. 4:**
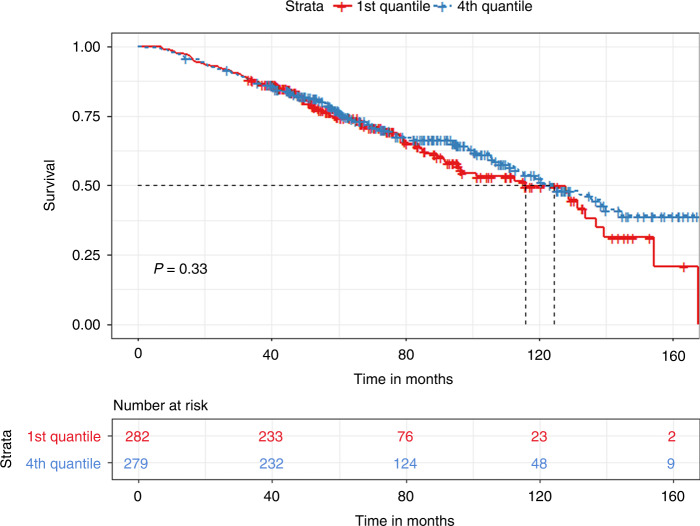
Kaplan Meier plot comparing OS between the 1st and the 4th quartile for TTT. Median OS, 115.8 vs. 124.4 months, *p* = 0.33.

The median TTT from the last chemotherapy date was 33 days (27–42 inter-quartile range). We divided the cohort based on the median TTT (33 days). We found that patients with a TTT of less than 33 days had significantly prolonged PFS (35.6 vs. 32.1 months, *p* < 0.03) but non-significant OS difference (128 vs. 122.2 months, *p* = 0.68) compared to patients with a TTT of more than 33 days **(**Figs. 1 and 2). When grouping patients based on inter-quartile TTT, we found that patients with a TTT of less than 27 days (1st quartile) had significantly prolonged PFS (36.7 vs. 30.9 months, *p* < 0.01) but non-significant OS differences (115.8 vs. 124.4 months, *p* = 0.33), compared to patients with a TTT of more than 42 days (4th quartile) **(**Figs. 3 and 4).

In univariate analysis, when compared to patients with a TTT of less than 33 days, those with more than 33 days had an HR = 1.17 (CI = 1.02–1.35, *p* < 0.03) for PFS. We then sought to perform a multivariable analysis to determine whether a prolonged TTT is an independent risk factor for shorter PFS in MM patients. Our model accounted for age, biochemical response prior to transplant, conditioning dosage, and the FISH result at diagnosis. We found that patients with prolonged TTT had an increased risk for shorter PFS, with an HR = 1.19 (CI = 1.02–1.39, *p* < 0.03). We then grouped patients into quartiles and compared the 1st quartile to the 4th quartile. The univariate analysis for PFS showed an HR = 1.37 (CI = 1.12–1.67, *p* < 0.01), and multivariable analysis showed an HR = 1.34 (CI = 1.08–1.66, *p* < 0.01) for patients in the 4th quartile of TTT.

**Fig. 5:**
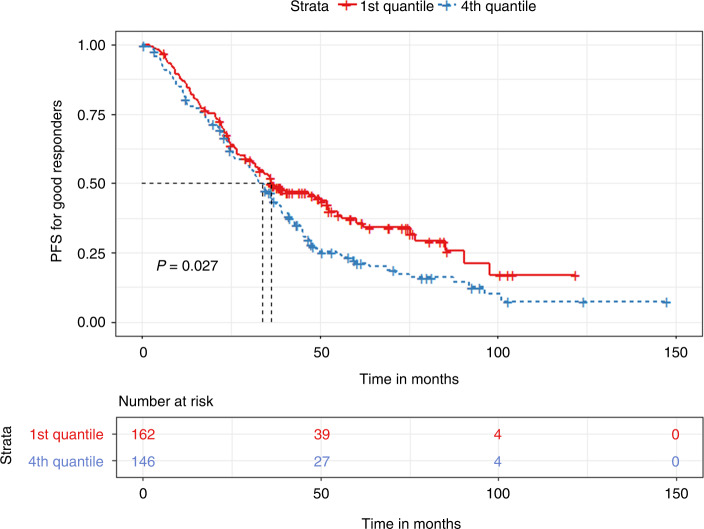
Kaplan Meier plot comparing PFS between 1st and 4th quartile for TTT in the Good responders group. Median PFS, 36.4 vs. 33.8 months, *p* < 0.03.

**Fig. 6:**
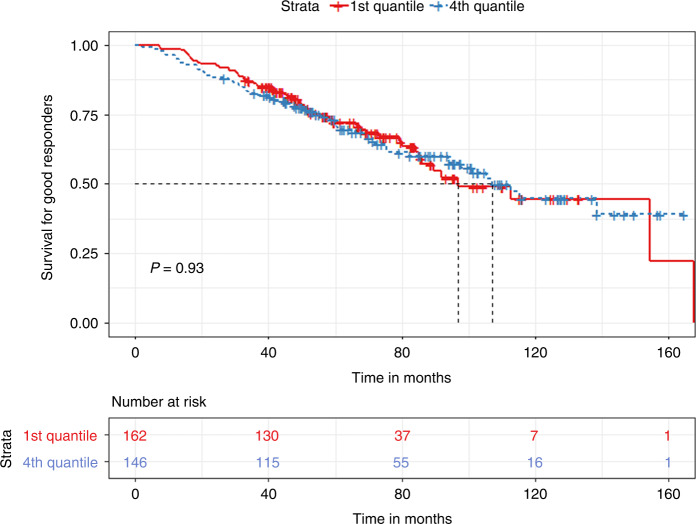
Kaplan Meier plot comparing OS between 1st and 4th quartile for TTT in the Good responders group. Median OS, 96.9 vs. 107.2 months, *p* = 0.93.

In a subgroup analysis based on the biochemical response achieved prior to transplant, we grouped patients into “good” responders (VGPR or better) and “bad” responders (less than VGPR). For the good responder group, we found non-significant differences based on median TTT in PFS and OS (36.4 vs. 37.3 months, *p* = 0.5 and 106.9 vs. 112.6 months, *p* = 0.81, respectively). In quartile comparisons, patients in the 1st quartile had significantly prolonged PFS (36.4 vs. 33.8 months, *p* < 0.03) compared to the 4th quartile group. For OS, no significant differences were found (96.9 vs. 107.2 months, *p* = 0.93) **(**Figs. 5 and 6).

**Fig. 7:**
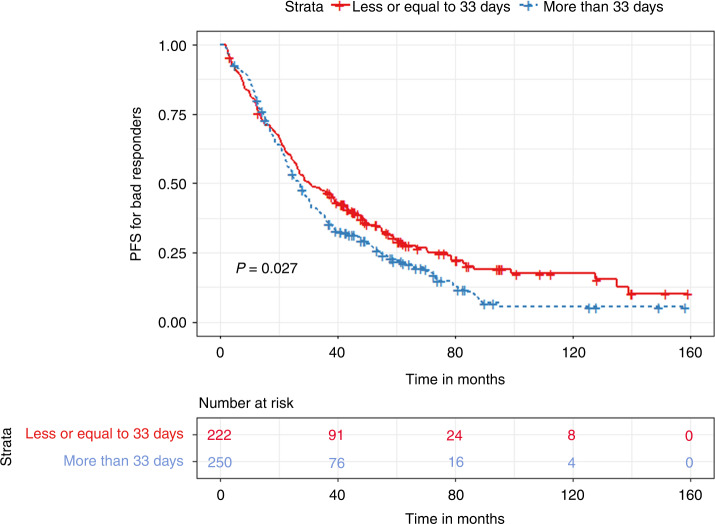
Kaplan Meier plot comparing PFS based on median TTT in the Bad responders group. Median PFS, 30.5 vs. 27 months, *p* < 0.03.

**Fig. 8:**
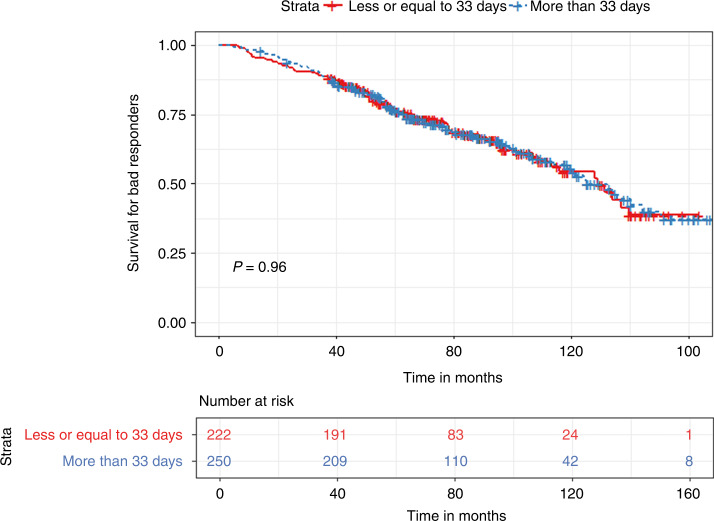
Kaplan Meier plot comparing OS based on median TTT in the Bad responders group. Median OS, 129 vs. 125 months, *p* = 0.96.

**Fig. 9:**
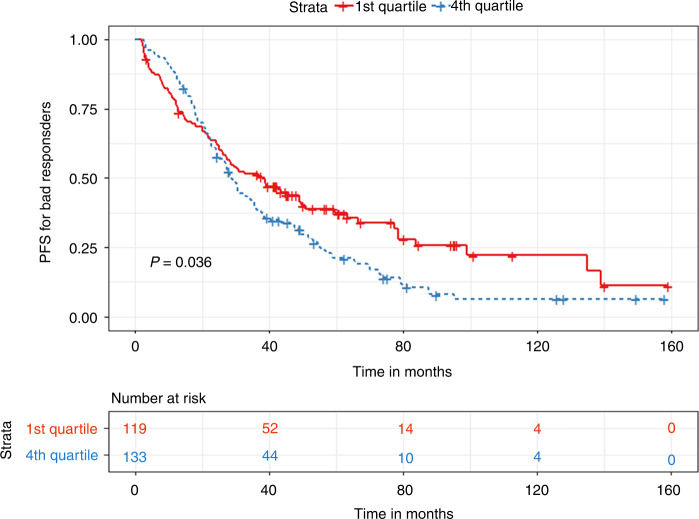
Kaplan Meier plot comparing PFS between 1st and 4th quartile for TTT in the Bad responders group. Median PFS, 37.7 vs. 28.7 months, *p* < 0.04.

**Fig. 10:**
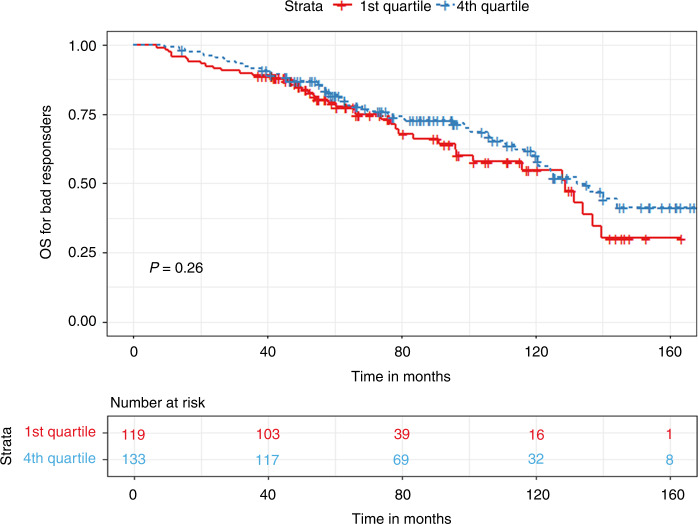
Kaplan Meier plot comparing OS between 1st and 4th quartile for TTT in the Bad responders group. Median OS, 129 vs. 132 months, *p* = 0.26.

In the bad responder group, patients with a TTT of less than 33 days had significantly prolonged PFS (30.5 vs. 27 months, *p* < 0.03) but similar OS (129 vs. 125 months, *p* = 0.96) compared to patients with a TTT of more than 33 days **(**Figs. 7 and 8). In terms of quartile comparison, patients in the 1st quartile had significantly prolonged PFS (37.7 vs. 28.7 months, *p* < 0.04) compared to the 4th quartile group. For OS, no significant differences were found (129 vs. 132 months, *p* = 0.26) **(**Figs. 9 and 10).

## Discussion

This is the first study to evaluate the impact of the time interval between the last chemotherapy date and the date of stem cell infusion on clinically relevant outcomes in newly diagnosed, transplant-eligible MM patients. In addition, we wanted to explore whether patients with underlying aggressive disease biology and early clonal re-emergence are disproportionally affected by TTT. We hypothesized that a prolonged TTT might be associated with inferior PFS, especially in patients with suboptimal responses to induction therapy.

Indeed, we showed that the time to transplant from the last chemotherapy date plays a significant role in the progression-free survival (PFS) of MM patients undergoing autologous stem cell transplant. With a median TTT of 33 days, we showed that patients with a protracted TTT (more than the median) had significantly worse PFS than patients with their stem cells infused in less than 33 days from the last chemotherapy date. Accordingly, patients in the 4th quartile of TTT had significantly worse PFS compared to patients in the 1st quartile. The detrimental effect was more pronounced when we grouped patients based on the IMWG biochemical response achieved at induction. More specifically, the most significant difference in PFS was observed in patients who did not achieve a better than PR response prior to transplant. This finding suggests that this cohort may particularly benefit from more intense chemotherapy regimens without prolonged treatment gaps in their disease course.

It is worth noting that the consistent PFS difference observed in our study did not translate into different outcomes for overall survival (OS). This was expected given the heterogeneity of our cohort since we included patients with significant differences in the induction treatment received. In addition, post-transplant maintenance was not routinely used for the majority of our cohort, and salvage treatment was not uniform. To date, TTT is not routinely reported in clinical studies, and most randomized control trials with NDMM transplant eligible patients report only the time to mobilization in the treatment protocols [18–20]. As a result of our findings, it might be beneficial for future trials to incorporate TTT in the assessment of patients, especially for those that show signs of relapse prior to stem cell infusion.

Our study has several limitations. Firstly, the retrospective nature of the study design lends itself vulnerable to its inherent biases and shortcomings. Secondly, as most patients did not have myeloma lab testing performed at the end of induction and immediately before transplant, we could not evaluate whether the difference observed in the PFS is indeed the result of disease progression during the chemotherapy-free period. This is an important aspect that should be explored in future studies since patients that show signs of relapse during this short drug-free period may comprise a unique cohort with very aggressive disease biology and inferior outcomes. Lastly, while our definition of CR required bone marrow testing in the pre-transplant evaluation, we did not report Minimal Residual Disease (MRD) assessment and thus cannot adjudicate whether TTT equally impacts patients that are MRD negative as the rest of the cohort.

Our study demonstrates the important role of TTT in clinical outcomes for NDMM patients. We showed that a prolonged TTT (more than 42 days) is associated with inferior outcomes compared to a tighter chemotherapy schedule (less than 27 days). This finding was especially prevalent in patients with less than VGPR at induction with retained prognostic significance in multivariable analysis. While further prospective studies are required to validate the impact of TTT on clinically relevant outcomes, we suggest that patients should not be given extensive chemotherapy-free periods prior to stem cell infusion, as this may adversely affect their disease course.

## Data Availability

The datasets generated during and/or analysed during the current study are available from the corresponding author on reasonable request.
